# Multi-model imaging of the interaction of nanomaterials with cells

**DOI:** 10.1107/S2052252518003548

**Published:** 2018-03-01

**Authors:** Jianwei Miao

**Affiliations:** a Department of Physics and Astronomy, and California NanoSystems Institute, University of California, Los Angeles, Los Angeles, CA 90095, USA

**Keywords:** nanomaterials, cellular imaging, coherent diffractive imaging, CDI, equal slope tomography, EST, scanning transmission X-ray microscopy, generalized Fourier iterative reconstruction, GENFIRE, multi-model imaging

## Abstract

A multi-model X-ray imaging approach was implemented to probe the interaction of nanomaterials with a mammalian cell in three dimensions. With further developments, this approach could have an impact on nanomedicine and nanotoxicology.

The development of functionalized nanomaterials has seen a stark increase in the last few decades. The scope of their application has also broadened dramatically. With this comes an increase in the possibility of human exposure to such materials and several of their properties give cause for concern (Nel *et al.*, 2006 ▸). Conversely, the potential applications in medicine as the next generation of therapeutics show great promise, with several nanodrug studies having currently lead to FDA approved products for cancer treatment (De Jong & Borm, 2008 ▸). What is clear is that at the cellular level, a better understanding of the mechanisms through which biological matter and nanomaterials interact is critical. Understanding these processes is imperative for the interpretation of therapeutic outcomes and resulting nanocarrier design iterations. In the context of toxicology, direct visualization of the sites and mechanism of action of nanomaterial-induced damage of cellular matter will play a key role in improving the biosafety of novel nanomaterials (Nel *et al.*, 2006 ▸).

Although existing techniques, such as fluorescence and electron microscopy, are frequently used, these methods provide an incomplete picture. Fluorescence microscopy can be lacking in context as unlabeled structures are invisible, whilst electron microscopy requires thin sectioning and has a limited field of view. To truly understand the nature of nanoparticle interactions with cellular matter it is important that these phenomena can be observed in as close to a physiological state as possible.

In this issue of **IUCrJ**, Jiang and collaborators (Yao *et al.*, 2018 ▸) implemented a multi-model imaging tool to probe the interaction of nanomaterials with a mammalian cell in three dimensions (Fig. 1 ▸). In the experiment, they first incubated and treated macrophages with Gd@C_82_(OH)_22_ nanoparticles, a promising antitumour agent. Dual-energy scanning transmission X-ray microscopy (STXM) was used to image a whole macrophage at different sample orientations (Kirz *et al.*, 1995 ▸). By tuning the X-ray energy below and above the Gd *M*_2_ absorption edge (*i.e*. 1186 and 1189 eV, respectively), they acquired two tomographic tilt series by rotating the sample around a single axis. Each tilt series consists of 46 images with a tilt range of ±79.4°.

The tilt series were reconstructed by an advanced algorithm, termed equal slope tomography (EST) (Miao *et al.*, 2005 ▸). EST is a Fourier-based iterative algorithm, which iterates between real and reciprocal space. In real space, physical constraints are enforced, including positivity (*i.e.* the electron density of the sample cannot be negative) and a loose support (*i.e.* a boundary larger than the three-dimensional envelope of the sample). In reciprocal space, the measured data are used as the constraint in each iteration. After several hundreds of iterations, the algorithm converges to a global solution that is consistent with the measured data and physical constraints. It has been experimentally demonstrated that EST not only produces superior results to other traditional tomographic algorithms, but also is broadly applicable to three-dimensional imaging of biological and physical samples, ranging from breast tumors and cellular structures to single atoms in materials (Lee *et al.*, 2008 ▸; Zhao *et al.*, 2012 ▸; Scott *et al.*, 2012 ▸).

By combining dual-energy STXM and EST, Jiang and collaborators reconstructed the macrophage structure with a three-dimensional resolution of 75–80 nm. Fig. 1 ▸(*a*) shows the three-dimensional volume rendering of the reconstructed macrophage with the Gd@C_82_(OH)_22_ nanoparticles in dark red, the nucleus in brown and different types of lysosomes in yellow. The three-dimensional reconstruction provides more faithful cellular structure information than the two-dimensional projection image (Fig. 1 ▸ *b*). Furthermore, by subtracting the two reconstructions measured above and below the Gd absorption edge, the three-dimensional distribution of the nanoparticles inside the macrophage was obtained (Fig. 1 ▸ *c*). They found that nanoparticles were mainly aggregated in lysosomes and no nanoparticles were distributed in the nucleus. These observations were further corroborated by the X-ray fluorescence microscopy image of the same macrophage (Fig. 1 ▸ *d*).

Looking forward, several additional developments could make this multi-model imaging approach widely applicable in nanomedicine and nanotoxicology. First, coherent diffractive imaging (CDI) has to be incorporated to improve the spatial resolution (Miao *et al.*, 1999 ▸, 2015 ▸). A particularly promising CDI method is ptychography (Rodenburg *et al.*, 2007 ▸; Thibault *et al.*, 2008 ▸), which has recently been applied to image the interaction of nanoparticles with mammalian cells (Gallagher-Jones *et al.*, 2017 ▸). Second, for imaging cellular structures, the resolution is ultimately limited by the radiation damage (Kirz *et al.*, 1995 ▸). Cryogenic techniques must be implemented to preserve the specimen and alleviate the radiation damage problem (Rodriguez *et al.*, 2015 ▸; Deng *et al.*, 2017 ▸). Another advantage of using cryo-preservation is the ability to trap samples at different stages of the nanoparticle–cell interaction. Third, a more powerful tomographic reconstruction algorithm than EST, termed GENeralized Fourier Iterative REconstruction (GENFIRE) (Yang *et al.*, 2017 ▸; Pryor *et al.*, 2017 ▸), can be incorporated into this multi-model imaging approach to improve the three-dimensional resolution. Finally, as both large-scale and tabletop coherent X-ray sources are rapidly advancing worldwide (Miao *et al.*, 2015 ▸), dedicated multi-model imaging beamlines and/or endstations, by taking advantage of X-ray brilliance, have to be implemented with user-friendly data acquisition, data analysis and image reconstruction software. With all these developments, the future of multi-model imaging of the bio–nano interface indeed looks bright.

## Figures and Tables

**Figure 1:**
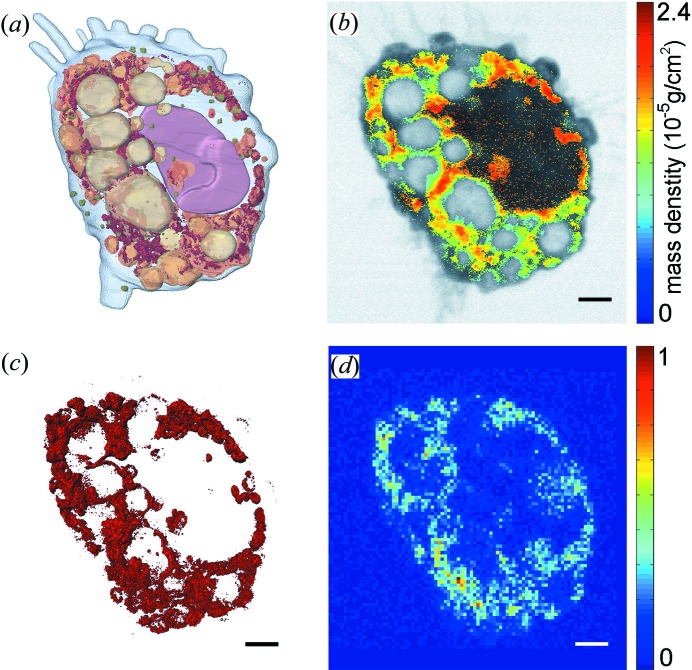
Multi-model imaging of the interaction of nanoparticles with a mammalian cell. (*a*) Three-dimensional image of a macrophage incubated and treated with Gd@C_82_(OH)_22_ nanoparticles, showing the nucleus (brown), various lysosomes (yellow) and the nanoparticle (dark red). The image was obtained by combining dual-energy X-ray microscopy with an advanced three-dimensional reconstruction algorithm, termed equal slope tomography (EST). (*b*) Distribution of the nanoparticles in a two-dimensional projection of the macrophage. (*c*) Three-dimensional intracellular distribution of the nanomaterials. (*d*) X-ray fluorescence microscopy image of the macrophage. The scale bar corresponds to 2.0 µm.
